# Effects of a Two-Year Home-Based Exercise Training Program on Oxidized LDL and HDL Lipids in Coronary Artery Disease Patients with and without Type-2 Diabetes

**DOI:** 10.3390/antiox7100144

**Published:** 2018-10-16

**Authors:** Sanna Tiainen, Antti Kiviniemi, Arto Hautala, Heikki Huikuri, Olavi Ukkola, Kari Tokola, Mikko Tulppo, Tommi Vasankari

**Affiliations:** 1 Sports Institute of Finland, 19100 Vierumäki, Finland; 2 Department of Health and Exercise and Paavo Nurmi Center, University of Turku, 20540 Turku, Finland; 3 Research of Internal Medicine, Medical Research Center Oulu, Oulu University, Hospital and University of Oulu, 90220 Oulu, Finland; Antti.M.Kiviniemi@oulu.fi (A.K.); heikki.huikuri@oulu.fi (H.H.); olavi.ukkola@oulu.fi (O.U.); mikko.tulppo@oulu.fi (M.T.); 4 Cardiovascular Research Group, Division of Cardiology, Oulu University Hospital, University of Oulu, 90220 Oulu, Finland; arto.hautala@hur.fi; 5 The UKK Institute for Health Promotion Research, 33500 Tampere, Finland; kari.tokola@uta.fi (K.T.); tommi.vasankari@uta.fi (T.V.)

**Keywords:** exercise, oxidized LDL lipids, oxidized HDL lipids, serum lipids

## Abstract

We investigated the effect of two-year home-based exercise training program on oxidized low-density lipoprotein LDL (ox-LDL) and high-density lipoprotein HDL (ox-HDL) lipids in patients with coronary artery disease (CAD), both with and without type-2 diabetes (T2D). Analysis of lipoprotein-oxidized lipids was based on the determination of baseline conjugated dienes in lipoprotein lipids. In order to study the effect of an exercise load on ox-LDL and ox-HDL lipids patients in both CAD and CAD + T2D intervention, groups were divided in three based on exercise load (high, medium, and low). During the two-year home-based exercise training program, the study showed that only higher training volume resulted in a decreased concentration of ox-LDL, while the two groups with lower training volumes showed no change. This result indicates that the training load needs to be sufficiently high in order to decrease the concentration of atherogenic ox-LDL lipids in patients with CAD and CAD + T2D. Interestingly, the concentration of ox-HDL did not change in any of the subgroups. This could indicate that the lipid peroxide-transporting capacity of HDL, suggested by results from exercise training studies in healthy adults, may not function similarly in CAD patients with or without T2D. Moreover, the lipid-lowering medication used may have had an influence on these results.

## 1. Introduction

Cardiovascular diseases (CVD) are a major global cause of premature death, chronic disability [1], and ischaemic heart disease, including coronary artery disease (CAD), which is the leading cause of death from CVD [2]. Major risk factors for cardiovascular diseases include hypertension, smoking, diabetes, diet factors, being overweight, obesity, physical inactivity, and elevated levels of serum total or LDL cholesterol, as well as low levels of HDL cholesterol [3,4,5,6]. Oxidized lipids in circulating LDL are also shown to be strongly associated with coronary atherosclerosis, arterial dysfunctions, and mortality [7,8,9,10]. In addition, several studies have demonstrated the relationship between oxidized LDL lipids (ox-LDL lipids) and CVD risk factors, such as hypertension [11], smoking [12], and obesity [13]. However, only a few studies have investigated the role of oxidized HDL lipids (ox-HDL lipids) in atherosclerosis and CVDs. One study observed that ox-HDL lipids are implicated in the risk of atherosclerosis, although in an opposite manner to that of ox-LDL lipids [14].

Physical activity and exercise training are key elements in the management of CAD [15,16] and type-2 diabetes (T2D) [17], while aerobic exercise has also shown to increase the likelihood of successful body-weight maintenance [18,19,20]. Both poor cardiorespiratory and muscular fitness, and low levels of leisure-time physical activity are also associated with higher concentrations of ox-LDL lipids [21]. However, both good cardiorespiratory fitness and muscular fitness seem to protect overweight subjects from an atherogenic lipid profile, such as a high level of ox-LDL lipids [22]. Earlier studies have also reported that acute exercise may induce an acute increase of ox-HDL lipids, suggesting that acute aerobic exercise may enhance the lipid peroxide-transporting capacity of HDL [23].

Recently, we have found that the concentration of ox-HDL lipids increased during a six-month aerobic exercise intervention, which suggests that aerobic exercise intervention may enhance the lipid peroxide-transporting capacity of HDL [24]. In the present study, we investigated the effect of a two-year home-based exercise training on ox-LDL and ox-HDL lipids in coronary artery disease patients with and without type-2 diabetes. 

## 2. Materials and Methods 

### 2.1. Subjects and Study Protocol

**Figure 1:**
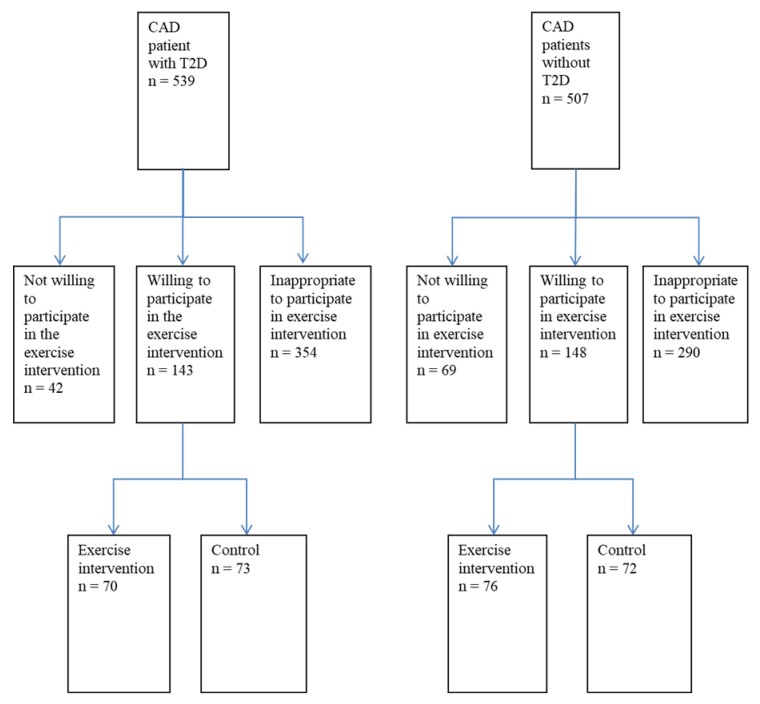
Patient selection protocol from the ARTEMIS database. Note: CAD, coronary artery disease; T2D, type-2 diabetes.

The ARTEMIS (innovation to reduce cardiovascular complications of diabetes at the intersection) study was initiated to assess the significance of autonomic, electrical, coronary angiographic, and metabolic markers in predicting cardiovascular events among CAD patients with (CAD + T2D patients) and without T2D (CAD patients) [25]. In addition to this, the study aimed to assess the prognostic significance of these markers in predicting CV events among CAD patients with and without T2D. CAD patients with and without T2D were recruited (1:1 matched in terms of age, sex, history of recent (<3 months) myocardial infarction and type of coronary intervention after angiography) from patients undergoing coronary angiography in the department of cardiology at Oulu University Hospital. As a substudy, both patients with and without T2D were chosen to undergo a two-year controlled exercise training trial with home monitoring to assess the effects of exercise training on risk profiles. Between August 2007 and March 2011, there were approximately 539 CAD patients with T2D and 507 CAD patients without T2D in the ARTEMIS database (Figure 1). Of those patients, 644 were deemed inappropriate to be included in the study due their failing to meet the selection criteria, while 111 were not willing to participate in the study. That resulted in a total of 291 patients who were eligible and willing to participate in the study. These 291 patients were divided into 2 groups consisting of an exercise training group (n = 146) and a control group (n = 145). The patients in each group were matched 1:1 in terms of gender and the presence of T2D. The 135 patients of the exercise group who took part in follow-up measurements were included in the analyses of cardiovascular risk factors according to the intention-to-treat principle. This study focused on levels of ox-LDL and ox-HDL lipids at the main research design (intervention vs. control), and during further analysis (results presented by exercise load and by waist circumference). The study was performed according to the Declaration of Helsinki. The local committee of research ethics of the Northern Ostrobothnia Hospital District approved the study protocol. All of the selected patients gave their written consent.

### 2.2. Exercise Training Intervention 

Patients in the exercise training group were given a training program for the study, along with a diary to record their training data (training mode, duration, and mean heart rate (Polar F1; Polar Electro Oy, Kempele, Finland). During the first 3 months of the study, the training program consisted of a weekly level of three 30 min endurance-based sessions (at a 50–60% intensity level) and a 30 min strength-based session. For the final 6 months, the weekly program consisted of five 40 min endurance-based sessions and one 30 min strength-based session. Of the five endurance-based sessions, 2 were at a 50–60% intensity level, 2 at a 60–70% intensity level, and 1 was interval training at a 70–80% intensity level. Patients in the control group did not receive any individually tailored exercise program.

### 2.3. Measurement of Leisure-Time Physical Activity (LTPA)

Patients were given a baseline health questionnaire about the frequency of their habitual LTPA. Based on this information, 4 physical activity groups were formed by modifying a scale originally developed by Saltin and Grimby [26].

(1) No LTPA (very little physical activity or light housework).
(2) Random LTPA (random light physical activity like walking or cycling).
(3) Moderate LTPA (Engages in physical activity at moderate level 2 to 3 times per week).
(4) Moderate or high LTPA (Engages in physical activity at moderate or high level more than 3 times per week).

The Saltin–Grimby Physical Activity-Level Scale has shown good validity [27] and has been shown to be related to both CV risk factors [27,28] and CV outcomes [29]. 

### 2.4. Exercise-Capacity Measurement

To assess exercise capacity, the patients were all asked to perform an incremental symptom-limited maximal exercise test on a bicycle ergometer (Monark Ergomedic 839 E; Monark Exercise AB, Vansbro, Sweden). The test was performed with a starting work rate level of 30 W. After that, the level was increased every 60 s by 15 W for male patients and 10 W for female patients. The test ended at voluntary exhaustion or ST depression 0.2 mV in electrocardiogram (ECG) (CAM-14; GE Healthcare, Freiburg, Germany). The maximal workload of the patients was calculated as the mean workload during the last minute of the test. Based on this maximal workload, maximal exercise capacity was then calculated.

### 2.5. Exercise Training Load

The weekly training load for the intervention group was calculated as the mean training impulse (TRIMP) using the following formula: TRIMP = ABC, in which A is the exercise time in minutes, B is the heart rate (proportioned to the heart rate reserve), and C is e^1·92B^ for men and e^1·67B^ for women [30]. TRIMP was used to divide the participants into 3 groups based on the exercise load (high, medium, and low training load).

### 2.6. Measurements of CV Risk Factors

Weight, waist, and hip measurements were taken to assess body composition. After a 10 min resting period, blood pressure was measured with the patients in a supine position. Blood samples were obtained after a 12 h overnight fast for analysis of blood lipids using standardized methods.

### 2.7. Determination of Oxidized Lipoprotein Lipids 

Examination of lipoprotein oxidized lipids was based on a determination of the baseline level of conjugated dienes in the lipoprotein lipids and it has been reported earlier in detail [31]. Appearance of conjugated dienes has traditionally been utilized as the index of oxidation in vitro and ex vivo examinations of LDL oxidation. First, serum LDL is isolated by precipitation with buffered heparin [31]. Isolation of the HDL part from serum samples is done using phosphotungstic acid precipitation [32]. The isolation methodology is validated for this purpose and it does not influence the level of oxidized lipids [31]. Lipids are removed from isolated lipoproteins by chloroform–methanol (2:1), dried under nitrogen, and thereafter redissolved in cyclohexane. The amount of peroxidized lipids in the lipoprotein lipids is evaluated by spectrophotometry as the amount of diene conjugation (AT 234 nm). Studies investigating assay validation have eliminated interference by nonspecific materials, and have demonstrated that diene conjugation is a measure of oxidative LDL modification that is found in all LDL lipid classes. Along with the particular absorption spectra at 234 nm, the existence of conjugated dienes has been confirmed by NMR studies [7]. The coefficient of variation for within-examination accuracy for the assurance of oxidized lipoprotein lipids was 4.4%, and the coefficient of variation for the between-examination accuracy was 4.5%.

### 2.8. Statistical Analysis 

First, the effect of the 2-year home-based exercise training intervention on ox-LDL and ox-HDL lipids was analyzed comparing intervention and control patients, both in coronary artery disease patients with and without type-2 diabetes. In order to study the effect of exercise load on ox-LDL and ox-HDL lipids, patients in both the CAD and CAD + T2D intervention groups were combined and the subjects were divided into 3 groups based on exercise load (high, medium, and low training load). Further, both in the CAD and in the CAD + T2D group, we divided the intervention group into 3 subgroups based on waist measurements. The men were divided into subgroups with waist measurements of <94, 94–102, and >102 cm. The women were divided into subgroups with waist measurements of <80, 80–88, and >88 cm. Descriptive group characteristics are shown as means with standard deviation (SD) or number of units (N) with percentages. Analysis of covariance (Ancova) adjusted for age, sex, and baseline value of outcome variable was used to estimate the effect of intervention and exercise on change in lipids. A *p*-value lower than 0.05 was considered statistically significant. All statistical analyses were conducted using IBM SPSS Statistics for Windows (Version 24.0: IBM Corp., Armonk, NY, USA).

## 3. Results

The baseline means of the plasma lipids between the CAD + T2D intervention and control group, and between the CAD intervention and the control group, did not differ in any of the measures before the study (Table 1), nor were there any changes during the intervention between the CAD + T2D intervention and control group, and between the CAD intervention and the control group.

### 3.1. Ox-LDL and Ox-HDL in Training Load Subgroups

**Figure 2:**
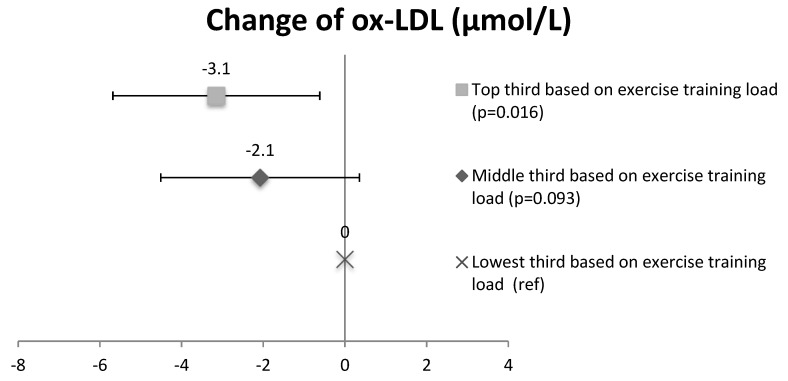
The change of oxidized LDL lipids in the intervention group (CAD and CAD + T2D) based on the exercise training load of the subgroups. Mean and confidence intervals, CI.

**Figure 3:**
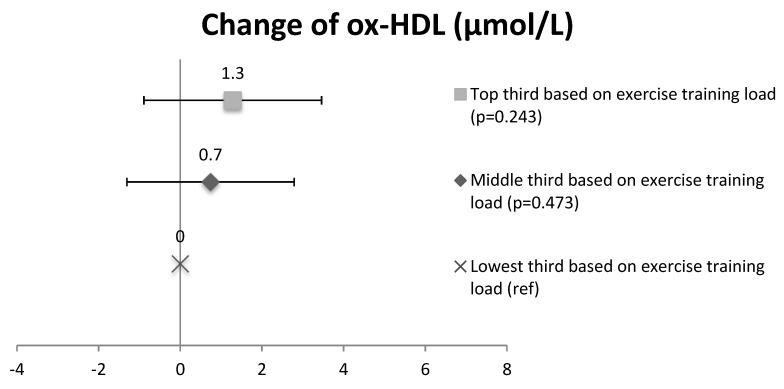
The change of oxidized HDL lipids in the intervention group (CAD and CAD + T2D) based on the exercise training load of the subgroups. Mean and CI.

In order to study the effect of training volume on ox-LDL and ox-HDL lipid changes, subjects in the intervention group (CAD and CAD + T2D patients combined) were divided into three subgroups based on the training load. The concentration of ox-LDL lipids in the subgroup with the high training load (41.3 ± 8.6 µmol/L at baseline and 39.4 ± 7.4 µmol/L at 2 years) decreased compared to the subgroup with a low training load (42.5 ± 9.3 µmol/L at baseline and 43.5 ± 10.4 µmol/L at 2 years) (the change, *p* = 0.016, Figure 2). No significant differences were seen between the medium training-load (44.9 ± 7.1 µmol/L at baseline and 43.5 ± 7.6 µmol/L at 2 years) and the low training-load subgroups in ox-LDL lipids (*p* = 0.093). In ox-HDL lipids, no significant differences were seen between the high training-load (28.8 ± 5.2 µmol/L at baseline and 32.0 ± 5.6 µmol/L at 2 years) and the low training-load subgroups (31.2 ± 5.9 µmol/L at baseline and 33.4 ± 7.3 µmol/L at 2 years) (*p* = 0.243) or between the medium training-load (30.6 ± 4.4 µmol/L at baseline and 33.3 ± 6.2 µmol/L at 2 years) and the low training-load subgroups (*p* = 0.473) (Figure 3).

### 3.2. Ox-LDL and Ox-HDL Lipids in the Intervention Waist Circumference Subgroups

**Figure 4:**
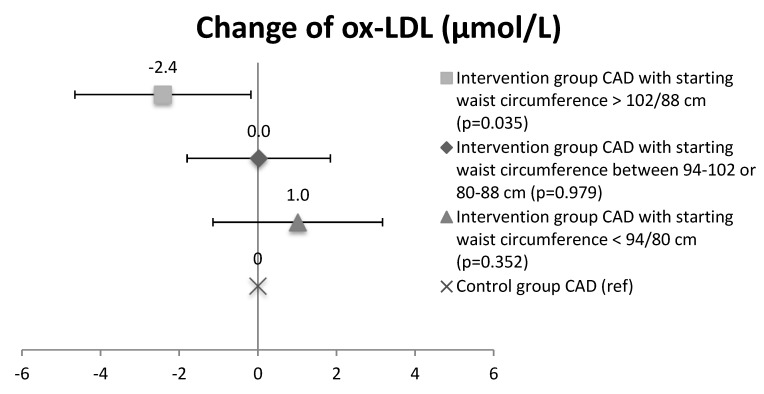
Change of oxidized LDL lipids in CAD waist-circumference subgroups. Mean and CI.

**Figure 5:**
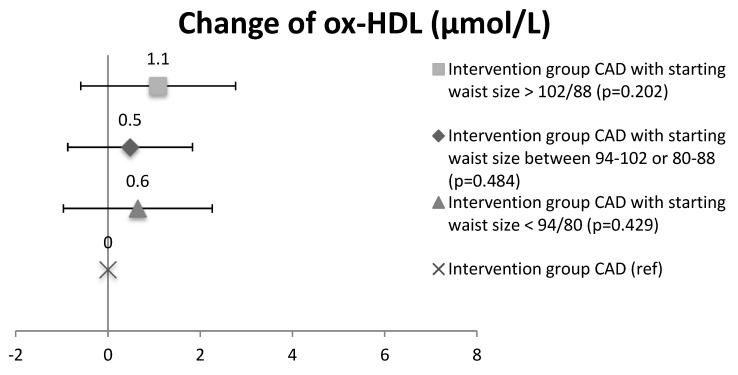
Change of oxidized HDL lipids in CAD waist-circumference subgroups. Mean and CI.

In CAD patients, the concentration of ox-LDL lipids decreased during intervention in the subgroups with the widest baseline waist circumference (>102 men/88 women: 45.9 ± 10.4 µmol/L at baseline and 43.1 ± 9.4 µmol/L at 2 years, n = 19) compared to the CAD control group (40.4 ± 9.7 µmol/L at baseline and 40.2 ± 10.0 µmol/L at 2 years; n = 65) (*p* = 0.035), while no significant changes were seen between other CAD waist-circumference subgroups (waist circumference 94–102 men/80–88 women: 42.4 ± 7.5 µmol/L at baseline and 42.0 ± 8.6 µmol/L at 2 years, n = 30; and waist circumference <94 men/<80 women: 40.9 ± 9.4 µmol/L at baseline and 41.5 ± 10.9 µmol/L at 2 years, n = 19) and controls (Figure 4). No changes were observed in ox-HDL lipids between CAD waist-circumference subgroups (waist circumference >102 men/88 women: 28.5 ± 4.8 µmol/L at baseline and 32.2 ± 5.7 µmol/L at 2 years, n = 19; waist circumference 94–102 men/80–88 women: 32.3 ± 3.6 µmol/L at baseline and 34.9 ± 4.2 µmol/L at 2 years, n = 30; and waist circumference <94 men/<80 women: 33.1 ± 5.7 µmol/L at baseline and 35.6 ± 5.9 µmol/L at 2 years n = 19) and controls (31.5 ± 4.1 µmol/L at baseline and 33.7 ± 4.5 µmol/L at 2 years; n = 65) (Figure 5), nor were there any changes seen in ox-LDL or ox-HDL lipids between CAD + T2D waist-circumference subgroups.

## 4. Discussion

The concentration of ox-LDL was decreased in the subgroup with the highest training load during the two-year home-based exercise training in patients with CAD and CAD + T2D. The present study showed that only the higher training volume resulted in a decreased concentration of ox-LDL, while the two groups with lower training volumes did not result in decreased concentration of ox-LDL. This result indicates that training load needs to be sufficiently demanding in order to decrease the concentration of atherogenic ox-LDL lipids patients with CAD and CAD + T2D. Our study is in line with earlier studies, where exercise training intervention decreased concentration of ox-LDL in subjects without CAD [33]. Interestingly, the concentration of ox-HDL was not changed even in the subgroup that performed the greatest volume of exercise. Therefore, although a six-month aerobic exercise intervention was shown to increase the concentration of ox-HDL lipids in healthy menopausal women [24], this was not the case in patients with CAD and CAD + T2D patients during this two-year home-based exercise training program. This could indicate that the lipid peroxide-transporting capacity of HDL, suggested by results from an exercise training study in healthy adults, may not similarly function in CAD patients with and without T2D. Moreover, the lipid-lowering medication used may have had an influence on these results, since it has been presented that statins may decrease the concentration of native LDL cholesterol, while it does not influence the concentration of oxidatively modified LDL particles [34]. However, nearly all participants used statins because all of them had CAD.

In this two-year controlled exercise training, we investigated the effects of home-based exercise training on oxidized LDL and HDL lipids in coronary artery disease patients with and without type-2 diabetes. When comparing the CAD and CAD + T2D intervention and control groups as a whole, we found no significant changes in the concentration of ox-HDL lipids. However, earlier studies have reported that a high volume of physical activity and exercise training are known to be valuable nonpharmacological means of managing CAD and T2D [15,16,17]. The exercise training intervention that resulted in a 19% increase in maximal oxygen uptake also decreased the concentration of ox-LDL in healthy adults [33]. Similarly, good aerobic fitness is associated with a low concentration of ox-LDL lipids [21]. However, this two-year home-based exercise training was not intensive enough to induce changes in serum lipids [25], and ox-LDL and ox-HDL lipids.

The CAD patients with the highest waist circumference at baseline reported significant changes in ox-LDL concentration. This finding is supported by an earlier study, where both good cardiorespiratory fitness and muscular fitness seemed to protect overweight subjects from atherogenic lipid profile, such as a high level of ox-LDL lipids [22]. Further, successful maintenance of weight loss has been reported to be accompanied by reduced ox-LDL lipids in obese men, which could indicate a decreased risk of atherosclerosis [13]. However, no statistically significant differences were seen between the waist-circumference subgroups in CAD + T2D patients. This could mean that the altered glucose metabolism in T2D patients might have influenced the ox-LDL lipid metabolism.

Earlier studies have reported that intense exercise, such as vigorous running, may induce an acute increase of ox-HDL [23]. Several studies have also indicated that acute and prolonged physical exercise decreases the concentration of ox-LDL lipids [33,35,36,37]. To our knowledge, no earlier studies have been published where the effects of exercise training on oxidized HDL and LDL lipids have been studied in CAD patients. Although the current study did not report significant changes in ox-HDL and ox-LDL lipids in all patients in the intervention group, high-volume training was accompanied with a decreased concentration of ox-LDL lipids. Therefore, physical activity and exercise training is considered to prevent atherogenic changes in lipids and to be an effective nonpharmacological method of preventing atherosclerosis.

## 5. Conclusions

This study shows that a two-year home-based exercise training program with a high volume is accompanied by a decreased concentration of oxidized LDL lipids. The concentration of oxidized HDL lipids did not change during intervention. Our results indicate that a high volume of physical activity and exercise training may protect even CAD patients from both an atherogenic lipid profile and a high level of oxidized LDL lipids; however, a lower volume of training may not have similar influence.

## Figures and Tables

**Table 1:** Clinical characteristics of the participants.

Measures	CAD and CAD + T2D Control Baseline (n = 126–132)	CAD and CAD + T2D Control 2 Years (n = 128–132)	CAD and CAD + T2D Exercise Intervention Baseline (n = 84–93)	CAD and CAD + T2D Exercise Intervention 2 Years (n = 85–93)
Age (years)	61.2 (6.1)	63.2 (6.1)	62.0 (5.2)	64.0 (5.2)
Gender, male (%)	98 (74%)	98 (74%)	72 (77%)	72 (77%)
Diabetics, n (%)	64 (50%)	64 (50%)	43 (46%)	43 (46%)
Smoking, n (%)	14 (10%)	18 (13%)	6 (7%)	8 (9%)
Height (cm)	172 (8)	172 (8)	171 (9)	171 (9)
Weight (kg)	83.6 (15.2)	84.0 (15.4)	82.1 (13.6)	81.4 (14.0)
BMI (kg/m^2^)	28.3 (3.9)	28.4 (4.1)	28.1 (3.7)	27.8 (3.7)
Waist (cm)	98.8 (12.9)	100.5 (13.6)	97.5 (11.2)	96.6 (11.1)
Systolic RR (mmHg)	145 (23)	145 (26)	145 (23)	144 (21)
Diastolic RR (mmHg)	82 (11)	80 (13)	83 (12)	80 (10)
HbA1c, %	6.5 (1.2)	6.2 (0.9)	6.2 (0.7)	6.0 (0.7)
Plasma glucose (mmol/L)	6.5 (1.7)	6.5 (1.6)	6.0 (1.1)	6.2 (1.2)
Total cholesterol (mmol/L)	4.0 (0.9)	4.0 (0.8)	4.0 (0.8)	4.1 (0.8)
Triglycerides (mmol/L)	1.4 (0.9)	1.5 (0.8)	1.5 (0.8)	1.5 (0.7)
Ox-HDL (µmol/L)	29.9 (4.6)	32.3 (4.9)	30.2 (5.1)	32.9 (6.2)
HDL cholesterol (mmol/L)	1.3 (0.3)	1.3 (0.3)	1.2 (0.3)	1.3 (0.3)
Ox-HDL/HDL-cholesterol	24.6 (6.5)	26.3 (7.1)	25.7 (7.3)	27.2 (8.9)
Ox-LDL (µmol/L)	41.8 (9.3)	41.2 (9.2)	43.1 (8.2)	42.1 (8.4)
LDL cholesterol (mmol/L))	2.3 (0.8)	2.3 (0.7)	2.3 (0.7)	2.4 (0.8)
Ox-LDL/LDL-cholesterol	19.9 (7.7)	19.9 (7.6)	20.0 (7.4)	19.4 (7.2)
Lipid-lowering medication, n (%)	119 (90%)	118 (90%)	84 (90%)	86 (93%)

Abbreviations: CAD: coronary artery disease; T2D: type 2 diabetes; BMI: body mass index; HDL: high density lipoprotein; LDL: low density lipoprotein; RR: blood pressure; HbA1c: glycated hemoglobin; Ox-HDL: oxidized HDL lipids; Ox-LDL: oxidized LDL lipids. The values denote mean (standard deviation) or number (%). Numbers of the participants differ somewhat between the measures.
